# Prevalence, associated factors and impact of mild cognitive impairment in hospitalized older adults with Parkinson’s disease: a cross-sectional study

**DOI:** 10.3389/fnagi.2025.1693417

**Published:** 2025-10-31

**Authors:** Siyuan Gong, Tianqi Wang, Rongzhu Tang, Wangjuan Hu, Wenjing Wang, Jia Li, Jihong Liu, Chunlian Liao

**Affiliations:** Department of Neurology, The Second Affiliated Hospital of Chongqing Medical University, Chongqing, China

**Keywords:** Parkinson’s disease, mild cognitive impairment, prevalence, associated factors, impact

## Abstract

**Background:**

Early identification of mild cognitive impairment (MCI) and timely interventions are essential to delay dementia in Parkinson’s disease (PD). This study aims to examine the prevalence of MCI among hospitalized older adults with PD, preliminarily identify related factors, and explore its possible clinical impact, with the goal of providing evidence to inform more targeted screening and intervention strategies.

**Methods:**

A cross-sectional survey was conducted in China. From July 2022 to January 2025, a total of 339 hospitalized older adults with PD were recruited from a hospital using convenience sampling. Data were collected on demographic characteristics, biochemical markers, and clinical assessments. MCI was evaluated using the Mini-Mental State Examination. Univariate analysis was conducted to examine potential associations between MCI and the collected variables. Multivariate logistic regression was then used to identify independent factors and their impact associated with MCI in hospitalized older adults with PD.

**Results:**

The results showed that the prevalence of MCI in hospitalized older adults with PD was 45.4%. Multivariate logistic regression analysis revealed that MCI in hospitalized older adults with PD was significantly associated with education level of primary and below [OR = 6.358, 95% CI (2.542, 15.902)] and junior [OR = 4.782, 95% CI (1.965, 11.635)], higher MDS-UPDRS-III scores [OR = 1.023, 95% CI (1.007, 1.039)], presence of anxiety [OR = 2.045, 95% CI (1.080, 3.873)], lower hemoglobin levels [OR = 0.983, 95% CI (0.968, 0.998)], and longer hospitalization duration [OR = 1.833, 95% CI (1.113, 3.017)].

**Conclusion:**

Our study observed a relatively high prevalence of MCI among hospitalized older adults with PD, and identified several associated factors, including lower educational level, greater severity of motor symptoms, anxiety, and reduced hemoglobin levels. These findings provide preliminary insights into factors that may warrant consideration when designing PD-MCI screening and intervention programs. Notably, we also found an association between PD-MCI and longer hospitalization duration, suggesting that early identification and management of MCI may help improve patient outcomes and reduce hospitalization burden.

## Introduction

With the aging of the global population, Parkinson’s disease (PD) and dementia have emerged as two major public health challenges (Padeiro et al., 2023). In China, the prevalence of PD among individuals aged 60 and above is 1.37%, which exceeds the global average of 1.05% for the same age group (Qi et al., 2021; Su et al., 2025). PD has thus become a significant health concern, severely affecting the wellbeing and quality of life of the older adult population in China (Xu et al., 2024). Cognitive impairment is one of the most common and disabling non-motor symptoms of PD (Aarsland et al., 2021). Depending on severity, it is categorized into mild cognitive impairment (MCI) and dementia (Aarsland et al., 2021). Approximately 1.705 million people globally are affected by Parkinson’s disease dementia (PDD), with notable regional differences in disease burden. East Asia accounts for the largest number of PDD cases—about 589,800—making it one of the most affected regions worldwide (GBD 2019 Dementia Collaborators, 2021). As the country with the highest number of PD patients in East Asia, China is facing a growing burden from PD-related complications (Zheng et al., 2023).

MCI is commonly regarded as an intermediate and potentially reversible stage between normal cognitive aging and dementia (Litvan et al., 2012). In PD patients, about 10–15% of those with MCI progress to dementia annually, and approximately 50% will develop dementia within 10–15 years (Wallace et al., 2022). This heterogeneity reflects the diverse neuropathological processes underlying MCI associated with PD (PD-MCI) (Knox et al., 2020; Paolini Paoletti et al., 2023), which may involve nigrostriatal dopaminergic neuronal loss as well as impairments in non-dopaminergic systems, including cholinergic, noradrenergic, and serotonergic pathways (Zarkali et al., 2024). In addition, genetic and molecular variations further modulate individual susceptibility to cognitive impairment in PD. Recent studies have identified that Alzheimer’s disease (AD)-related risk genes, such as WW domain-containing oxidoreductase (WWOX) and MAF bZIP transcription factor (MAF), significantly affect language, memory, and executive function in PD patients, suggesting overlapping molecular mechanisms between AD and PD in cognitive dysfunction (Yuan et al., 2025). Moreover, glucocerebrosidase (GBA) gene mutations exacerbate cognitive decline in PD by disrupting lysosomal function and *α*-synuclein metabolism, leading to toxic protein aggregation and impaired synaptic plasticity (Chang et al., 2024). As cognition deteriorates, patients often develop hallucinations, psychiatric symptoms, and motor complications, which ultimately impair functional independence and reduce quality of life (Gallagher et al., 2024; Weintraub et al., 2022). PDD is also associated with severe physical disability, frequent hospitalizations, and an increased risk of mortality (Benito-Rodríguez et al., 2025; Szeto et al., 2020).

Early identification of MCI and the implementation of timely pharmacological and non-pharmacological interventions are therefore essential to delay the onset of dementia in PD. Previous studies have indicated that up to 40% of PD patients may experience MCI (Baiano et al., 2020). Hospitalized PD patients, in particular, may face a heightened risk of cognitive impairment due to medication changes, infections, disrupted sleep–wake cycles, and multiple comorbidities (Deng et al., 2024; Tönges et al., 2024). However, PD-MCI often goes undetected in routine clinical settings because it does not significantly interfere with functional independence. Although existing studies have explored the prevalence and risk factors of PD-MCI, most have focused on community-based or outpatient populations (Bäckström et al., 2022; Domellöf et al., 2015; Peraza et al., 2017; Yarnall et al., 2014), with relatively limited attention paid to hospitalized patients. Given their higher vulnerability, it is critical to investigate the prevalence, associated factors, and clinical implications of PD-MCI in hospitalized older adults. Therefore, this study aims to examine the prevalence of MCI among hospitalized older adults with PD, preliminarily identify related factors, and explore its possible clinical impact, with the goal of providing evidence to inform more targeted screening and intervention strategies. We hypothesized that (1) the prevalence of MCI would be relatively high among hospitalized older adults with PD, and (2) specific factors such as lower educational level, greater motor symptom severity, and comorbidities would be associated with an increased risk of PD-MCI.

## Methods

### Study design

This descriptive, observational, and cross-sectional study was conducted in accordance with the Strengthening Reporting of Observational Studies in Epidemiology (STROBE) guidelines (Von Elm et al., 2007) (Supplementary Table S1).

### Participants

The study was conducted using a convenience sampling method and the participants were hospitalized older adults with PD from July 2022 to January 2025 in the Department of Neurology of the Second Affiliated Hospital of Chongqing Medical University. The inclusion criteria were as follows: (a) Met the clinical diagnostic criteria for PD established by the Movement Disorder Society (MDS) (Litvan et al., 2012); (b) Aged≥60 years; (c) Hospitalized patients; (d) Able to provide informed consent. The exclusion criteria were as follows: (a) Met the diagnostic criteria for PDD proposed by the MDS Task Force (Emre et al., 2007); (b) Diagnosed with other neurodegenerative diseases in addition to PD (especially multiple system atrophy, progressive supranuclear palsy, or dementia with Lewy bodies); (c) Cognitive impairment caused by other factors (e.g., seizures, stroke, or traumatic brain injury); (d) Long-term use of anticholinergic medications or sedatives; e. Unable to complete clinical scale assessments or blood sample collection.

### Sample size

According to the sample size estimation principle proposed by Bacchetti and Leung (2002), the baseline sample size should meet the criterion of Nbase = k × n (k∈[5,10]). In this study, a total of 27 independent variables were included. Taking into account a potential 20% attrition rate, the minimum required sample size was calculated to be 169 participants. Ultimately, a total of 339 participants were included in the study.

### Instruments

#### General questionnaire

A general questionnaire was developed by the researchers to collect relevant data for the study, including participants’ demographics information and biochemical markers. Demographics information included age, sex, educational level, body mass index (BMI), disease duration, smoking history, alcohol consumption history, history of hypertension, history of diabetes, levodopa equivalent daily dose (LEDD), and duration of hospitalization. Biochemical markers included serum creatinine (SCr), serum uric acid (SUA), hemoglobin (Hb), albumin (Alb), hemoglobin A1c (HbA1c), triglycerides (TG), total cholesterol (TC), high-density lipoprotein cholesterol (HDL-C), and low-density lipoprotein cholesterol (LDL-C).

#### Barthel index (BI)

The Barthel Index (BI), developed by Mahoney and Barthel (1965), is a widely used assessment tool for evaluating individuals’ ability to perform activities of daily living (ADL). The scale consists of 10 items, each scored based on the level of independence in performing a specific task. The total score ranges from 0 to 100, with higher scores indicating greater independence. A score below 40 suggests severe functional impairment, scores between 40 and 60 indicate moderate impairment, and scores above 60 reflect mild impairment. The Cronbach’s alpha coefficient of the BI ranges from 0.70 to 0.96, indicating good internal consistency.

#### Hoehn and Yahr stages

The Hoehn and Yahr (H-Y) staging scale, proposed by Hoehn and Yahr (1967), is a clinical classification system used to assess the severity of PD. It categorizes patients into different stages based on the presence and extent of motor symptoms and functional impairment.

#### Movement disorder society-unified Parkinson’s disease rating scale

The Movement Disorder Society–Unified Parkinson’s Disease Rating Scale (MDS-UPDRS), revised by the Movement Disorder Society in 2008 (Goetz et al., 2008), is a comprehensive tool for assessing the severity of PD. Each item is rated on a scale from 0 (normal) to 4 (severe), with higher scores indicating more severe symptoms. The scale demonstrates excellent internal consistency, with a total Cronbach’s alpha coefficient ranging from 0.79 to 0.93, and shows a high correlation (r = 0.96) with the original UPDRS (Goetz et al., 2008). In this study, MDS-UPDRS-I was used to evaluate non-motor symptoms, MDS-UPDRS-II to assess activities of daily living, and MDS-UPDRS-III (off-state) to assess motor symptoms. Patients discontinued levodopa-containing preparations for 12 h and dopamine receptor agonists for 72 h prior to the evaluation to ensure that they were in a defined “off” state.

#### Hamilton anxiety rating scale

The Hamilton Anxiety Rating Scale (HAMA), developed by Hamilton (1959), is widely used for the diagnosis of anxiety disorders, monitoring treatment outcomes, and in clinical research. The scale consists of 14 items covering both somatic and psychological symptoms of anxiety. The total score ranges from 0 to 56, with higher scores indicating greater severity of anxiety symptoms. A cut-off score of 14 is commonly used in international studies to indicate clinically significant anxiety. The scale has demonstrated good internal consistency, with a Cronbach’s alpha coefficient ranging from 0.74 to 0.92.

#### Hamilton depression rating scale

The 17-item Hamilton Depression Rating Scale (HAMD-17), developed by Hamilton (1960), is one of the most widely used instruments for assessing the severity of depressive symptoms and distinguishing depressed patients from non-depressed individuals. Each item is rated on a scale from 0 (absent) to 4 (severe), with total scores ranging from 0 to 56. A total score below 7 is generally considered within the normal range, while higher scores indicate more severe depressive symptoms. The scale has shown good internal consistency, with a Cronbach’s alpha coefficient ranging from 0.76 to 0.88.

#### Mini-mental state examination (MMSE)

The Mini-Mental State Examination (MMSE), developed by Folstein et al. (1975) in 1975, is one of the cognitive screening tools recommended by the Movement Disorder Society. The scale consists of 30 items assessing orientation, memory, attention, language, and visuospatial abilities. Total scores range from 0 to 30, with higher scores indicating better cognitive function. The internal consistency of the MMSE is acceptable, with a Cronbach’s alpha coefficient ranging from 0.80 to 0.90. In this study, MCI was defined based on MMSE scores adjusted for education level: ≤19 for illiterate individuals, ≤22 for those with primary school education, and ≤26 for those with junior high school education or above (Li et al., 2016).

### Data collection

Before initiating the study, all research team members received standardized training to ensure accurate data collection. Prior to the formal survey, a designated team member explained the purpose, significance, and key points of the study to each participant using a standardized script. Participants were informed of their right to withdraw from the study at any time without any consequences. After obtaining written informed consent, the formal survey was conducted. Upon completion, two research team members reviewed each questionnaire on-site to check for missing items or obvious logical errors. If issues were identified, they clarified them with the participant and corrected the questionnaire accordingly. Additional data on patients’ biochemical markers were retrieved from the electronic medical record system, and hospitalization duration was recorded upon discharge.

### Ethics statement

This study was conducted in accordance with the Declaration of Helsinki and its later amendments, or comparable ethical standards. Ethical approval was obtained from the Ethics Committee of the Second Affiliated Hospital of Chongqing Medical University (Approval no. 2022:110). Participants were assured that all responses would remain anonymous and that their personal information and survey data would be kept strictly confidential. Written informed consent was obtained from all participants.

### Statistical methods

Research data were entered into WPS Office Excel 2025 by two independent researchers for accuracy verification, and all statistical analyses were performed using IBM SPSS Statistics version 27.0. For quantitative data, normally distributed variables were presented as mean ± standard deviation (SD), while non-normally distributed variables were reported as median and interquartile range (IQR). Categorical variables were described using frequencies and percentages. Univariate analyses were conducted using the *t*-test, Mann–Whitney *U* test, or chi-square test to examine differences in demographics information, biological markers, and clinical assessments between hospitalized older adults with PD with and without MCI. Variables showing statistically significant differences in univariate analyses (*p <* 0.05) were entered into a multivariate binary logistic regression model to identify independent factors associated with MCI. The logistic regression analysis was performed using the backward selection method. Adjusted odds ratios (ORs) with 95% confidence intervals (CIs) were calculated for each independent variable to quantify the strength of association. Model fit was assessed using the Hosmer–Lemeshow goodness-of-fit test, and a *p*-value < 0.05 was considered statistically significant.

## Results

### Characteristics of the participants and differences in MCI

A total of 394 hospitalized older adults with PD met the inclusion and exclusion criteria and were invited to participate in this study. Of these, 339 patients were ultimately enrolled. Fifty-five patients were excluded due to refusal to participate (*n* = 37) or missing biochemical marker data (*n* = 18). Among the 339 participants, MCI was identified in 45.4% (154/339). The participants ranged in age from 60 to 87 years, with a median age of 70. The largest proportion of participants by characteristic were female (52.8%), had an education level of junior (36.6%), and had a disease duration of 5 years or less (77.3%). Most participants had no history of smoking (82.6%), drinking (87.3%), hypertension (63.1%), or diabetes (78.8%). The levodopa equivalent daily dose (LEDD) ranged from 300.00 to 512.50 mg/day, with a median of 375 mg/day. The median length of hospitalization duration was 6 days, and 39.2% of patients had a longer hospitalization duration (greater than the median) (Pezeshkian et al., 2025). Additional participant characteristics are presented in Table 1.

**Table 1:** Characteristics of the participants.

Variables	Total (*n* = 339)	Non-MCI (*n* = 185)	MCI (*n* = 154)	*P*
Demographics information				
Age [years, M (P25, P75)]	70 [66, 76]	70 [67, 76]	71 [65, 76]	0.917
Sex *n* (%)				**0.011** ^**^
Male	160 (47.2%)	99 (53.5%)	61 (39.6%)	
Female	179 (52.8%)	86 (46.5%)	93 (60.4%)	
Education level *n* (%)				**<0.001** ^***^
Primary and below	106 (31.1%)	42 (22.7%)	64 (41.6%)	
Junior	124 (36.6%)	59 (31.9%)	65 (42.2%)	
High or trade school	60 (17.7%)	43 (23.2%)	17 (11.0%)	
Junior college and above	49 (14.5%)	41 (22.2%)	8 (5.2%)	
BMI [kg/m^2^, M (P25, P75)]	23.19 [21.26, 25.71]	23.44 [21.56, 25.78]	22.79 [20.80, 25.39]	0.066
Disease duration (years) *n* (%)				0.051
≤5	262 (77.3%)	150 (81.1%)	112 (72.7%)	
5 ~ 10	61 (18.0%)	30 (16.2%)	31 (20.1%)	
>10	16 (4.7%)	5 (2.7%)	11 (7.1%)	
History of smoking *n* (%)				0.817
Yes	59 (17.4%)	33 (17.8%)	26 (16.9%)	
No	280 (82.6%)	152 (82.2%)	128 (83.1%)	
History of drinking *n* (%)				0.615
Yes	43 (12.7%)	25 (13.5%)	18 (11.7%)	
No	296 (87.3%)	160 (86.5%)	136 (88.3%)	
History of Hypertension *n* (%)				0.279
Yes	125 (36.9%)	73 (39.5%)	52 (33.8%)	
No	214 (63.1%)	112 (60.5%)	102 (66.2%)	
History of Diabetes *n* (%)				0.938
Yes	72 (21.2%)	39 (21.1%)	33 (21.4%)	
No	267 (78.8%)	146 (78.9%)	121 (78.6%)	
LEDD [mg/d, M (P25, P75)]	375 [300.00, 512.50]	375 [300.00, 507.89]	399.50 [300.00, 516.88]	0.106
Longer hospitalization duration *n* (%)				**0.002** ^**^
Yes	133 (39.2%)	59 (31.9%)	74 (48.1%)	
No	206 (60.8%)	126 (68.1%)	80 (51.9%)	
Biochemical markers				
SCr [μmol/L, M (P25, P75)]	66.3 [55.4, 78.8]	65.60 [55.10, 77.85]	66.65 [55.78, 79.95]	0.401
SUA [μmol/L, M (P25, P75)]	281.0 [225.0, 331.0]	279.40 [219.90, 327.25]	287.15 [227.75, 345.00]	0.469
Hb [g/L, M (P25, P75)]	129.0 [119.0, 138.0]	131.00 [122.00, 140.00]	127.00 [115.75, 135.00]	**<0.001** ^***^
Alb [g/L, M (P25, P75)]	41.1 [38.8, 43.8]	41.1 [38.70, 43.65]	41.6 [38.88, 43.83]	0.503
HbA1c [%, M (P25, P75)]	5.80 [5.40, 6.10]	5.80 [5.50, 6.10]	5.70 [5.40, 6.20]	0.564
TG [mmol/L, M (P25, P75)]	1.02 [0.74, 1.37]	0.99 [0.75, 1.37]	1.07 [0.72, 1.44]	0.389
TC (mmol/L, $\bar{x}$ ±s)	4.43 ± 1.09	4.35 ± 1.13	4.51 ± 1.03	0.112
HDL-C [mmol/L, M (P25, P75)]	1.32 [1.13, 1.53]	1.33 [1.13, 1.55]	1.32 [1.13, 1.50]	0.582
LDL-C [mmol/L, M (P25, P75)]	2.38 [1.79, 2.92]	2.38 [1.73, 2.89]	2.37 [1.85, 2.98]	0.340
Clinical assessment				
Barthel index *n* (%)				0.192
<40	305 (90.0%)	170 (91.9%)	135 (87.7%)	
40 ~ 60	33 (9.7%)	15 (8.1%)	18 (11.7%)	
>60	1 (0.3%)	0 (0%)	1 (0.6%)	
Hoehn-Yahr stages *n* (%)				**0.006** ^**^
I	48 (14.2%)	33 (17.8%)	15 (9.7%)	
II	154 (45.4%)	86 (46.5%)	68 (44.2%)	
III	94 (27.7%)	50 (27.0%)	44 (28.6%)	
IV	27 (8.0%)	11 (5.9%)	16 (10.4%)	
V	16 (4.7%)	5 (2.7%)	11 (7.1%)	
MDS-UPDRS-I [M (P25, P75)]	9.0 [5.0, 14.0]	8.0 [5.0, 12.0]	10.0 [6.0, 16.0]	**0.006** ^**^
MDS-UPDRS-II [M (P25, P75)]	11.0 [8.0, 16.0]	10.0 [7.0, 14.0]	13.0 [8.0, 20.0]	**0.001** ^**^
MDS-UPDRS-III [M (P25, P75)]	32.0 [22.0, 44.0]	29.0 [20.0, 39.0]	38.0 [26.0, 51.0]	**<0.001** ^***^
Anxiety				**<0.001** ^***^
Yes	61 (18.0%)	21 (11.4%)	40 (26.0%)	
No	278 (82.0%)	164 (88.6%)	114 (74.0%)	
Depression				**0.029** ^*^
Yes	185 (54.6%)	91 (49.2%)	94 (61.0%)	
No	154 (45.4%)	94 (50.8%)	60 (39.0%)	

Data are presented as *n* (%) or Median (Interquartile Range), M (P25, P75), or Mean±SD, $\bar{x}$ ± s. *p < 0.05, **p < 0.01, ***p < 0.001. The bolding was originally used to visually highlight data with *p* < 0.05. We believe it is acceptable to remove the bold formatting, as the footnote sufficiently indicates statistical significance. Scr, Serum creatinine; SUA, Serum uric acid; Hb, Hemoglobin; Alb, Albumin; HbA1c, Hemoglobin A1c; TG, Triglyceride; TC, Total cholesterol; HDL-C, Highdensity lipoprotein cholesterol; LDL-C, Lowdensity lipoprotein cholesterol.

### Univariate analysis of MCI in hospitalized older adults with PD

Univariate analysis identified several variables potentially associated with MCI in hospitalized older adults with PD (*p <* 0.05). These included sex (*p =* 0.011), education level (*p <* 0.001), Hoehn-Yahr stage (*p =* 0.006), MDS-UPDRS-I (*p =* 0.006), MDS-UPDRS-II (*p =* 0.001), and MDS-UPDRS-III (*p <* 0.001), anxiety (*p <* 0.001), depression (*p =* 0.029), Hb (*p <* 0.001), and longer hospitalization duration (*p =* 0.002) (Table 1).

### Multivariate logistic regression analysis of factors associated with MCI and its impact in hospitalized older adults with PD

**Figure 1:**
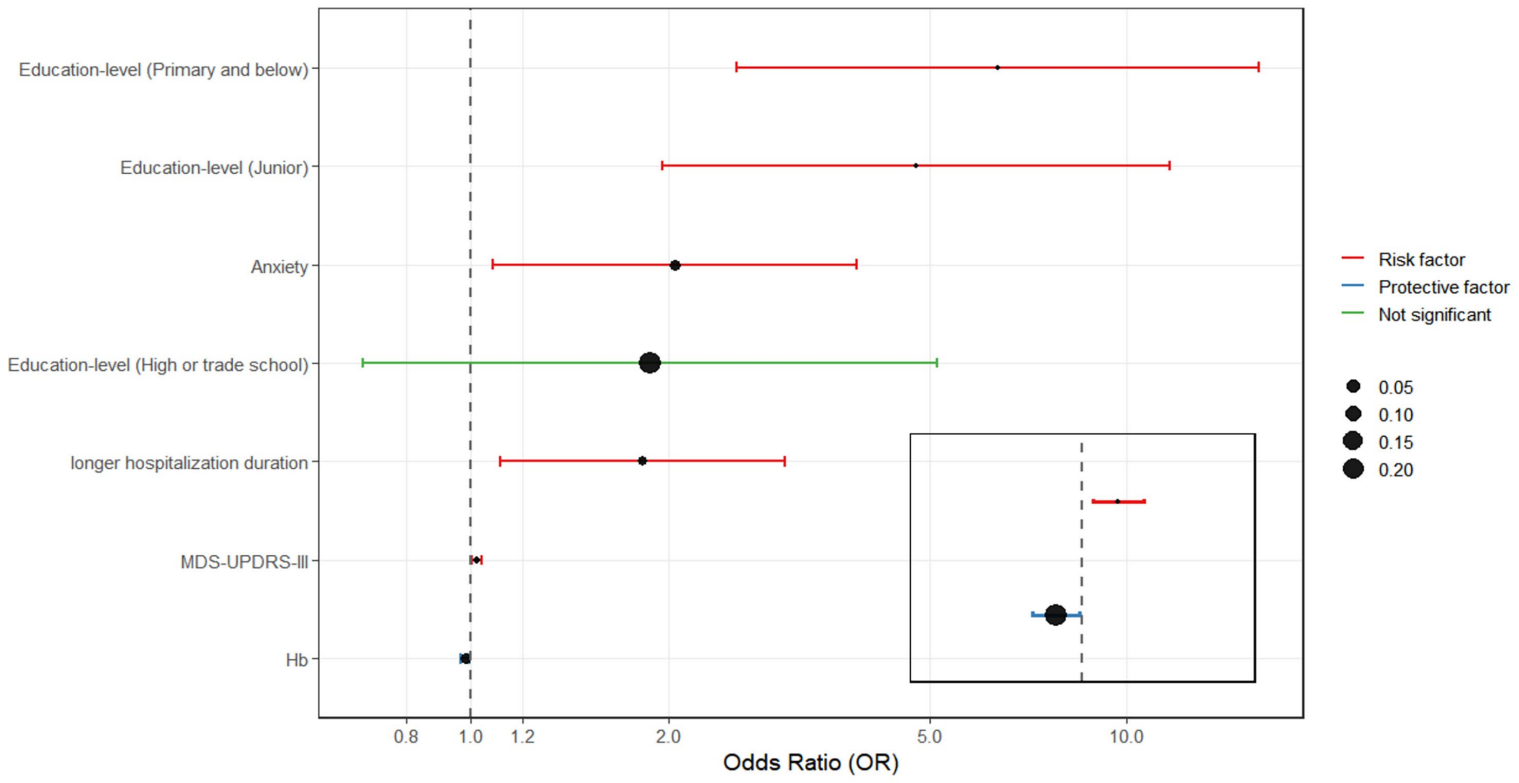
Odds ratios (OR) and 95% confidence intervals (95% CI) for factors that are relation to MCI.

A multivariate logistic regression analysis was performed using the backward selection method, with MCI as the dependent variable. Independent variables included those found to be statistically significant in the univariate analysis (*p <* 0.05). The results indicated that MCI was significantly associated with education level of primary and below [OR = 6.358, 95% CI (2.542, 15.902)] and junior [OR = 4.782, 95% CI (1.965, 11.635)]. Other independent risk factors included higher MDS-UPDRS-III scores [OR = 1.023, 95% CI (1.007, 1.039)], presence of anxiety [OR = 2.045, 95% CI (1.080, 3.873)], lower hemoglobin levels [OR = 0.983, 95% CI (0.968, 0.998)], and longer hospitalization duration [OR = 1.833, 95% CI (1.113, 3.017)] (Table 2 and Figure 1). The result of the Hosmer-Lemeshow test showed that χ^2^ = 4.075, *p =* 0.850 > 0.05, and the R^2^ = 0.256.

**Table 2:** Multivariate logistic regression analysis of factors influencing MCI in hospitalized older adults with PD (*n* = 339).

Variables	Regression coefficient	Standard error	Wald	*p*-value	aOR	95% CI	
						Upper limit	Lower llimit
Constant	−0.460	1.140	0.163	0.686	0.631		
Education level (refer to junior college and above)							
Primary and below	1.850	0.468	15.637	< 0.001	6.358	2.542	15.902
Junior	1.565	0.454	11.899	0.001	4.782	1.965	11.635
High or trade school	0.631	0.513	1.511	0.219	1.879	0.687	5.136
MDS-UPDRS-III	0.022	0.008	7.933	0.005	1.023	1.007	1.039
Anxiety	0.715	0.326	4.823	0.028	2.045	1.080	3.873
Hb	−0.017	0.008	4.627	0.031	0.983	0.968	0.998
Longer hospitalization duration	0.606	0.254	5.667	0.017	1.833	1.113	3.017

## Discussion

Although previous studies have examined cognitive function in older adults and individuals with PD, there is limited research specifically addressing the prevalence of MCI in hospitalized older adults with PD. To our knowledge, this is the first preliminary exploration in China to investigate the prevalence, associated factors, and impact of MCI in this population.

Our findings indicate that 45.4% of hospitalized older adults with PD in our sample had MCI—a prevalence notably higher than that reported in most previous studies. For instance, Marras et al. (2013) reported a prevalence of 33% among hospitalized PD patients in a cross-sectional study. Two cohort studies reported MCI rates of 39.6% (Monastero et al., 2018) and 28.9% (Pedersen et al., 2017), respectively. Community-based studies have shown MCI prevalence rates of 43.0% (Bäckström et al., 2022) in a cross-sectional analysis and 42.6 and 42.5% in two cohort studies (Domellöf et al., 2015; Yarnall et al., 2014). By comparison, the prevalence among outpatient PD populations is lower at 28.7% (Peraza et al., 2017). These variations may stem from differences in the study populations, as earlier studies often included PD patients across a broad range of ages and settings. Aging itself is a well-established risk factor for MCI. Older adults are more susceptible to cognitive decline due to age-related changes such as hippocampal atrophy, imbalance in amyloid protein metabolism, increased inflammatory responses, and the vulnerability of memory-related neurons (Das et al., 2019; Ma et al., 2025). Compared with community-dwelling or outpatient PD patients, hospitalized older adults with PD may face an elevated risk of cognitive impairment due to factors such as poor symptom control, infections, sleep disturbances, medication adjustments, and multiple comorbidities (Aminoff et al., 2011; Tönges et al., 2024). Notably, the prevalence of MCI among hospitalized older adults with PD in this study was significantly higher than both the reported rate of 14.7% in the general Chinese older adult population (Xue et al., 2018) and the global pooled prevalence of 21.2% (Chen et al., 2023). This highlights the unique neurodegenerative pathology of PD and its additional impact on cognitive function. PD is characterized by the early loss of dopaminergic neurons in the substantia nigra and the accumulation of misfolded alpha-synuclein proteins in Lewy bodies (Poewe et al., 2017). These pathological changes affect cognition, sensory processing, and motor function through complex interactions with postsynaptic neurons (Kulkarni et al., 2022). The decline in striatal dopamine levels impairs key cognitive domains such as working memory, visuospatial skills, and attention (Westbrook et al., 2020). Furthermore, cerebral microvascular damage, white matter lesions, and neurotransmitter dysfunction may compound the deterioration in cognitive performance (Brandão et al., 2020). Given these findings, early and systematic cognitive screening for hospitalized older adults with PD is of critical clinical importance. Identifying MCI at an early stage allows for timely interventions that may slow disease progression, support the development of individualized care strategies, and ultimately improve patient outcomes.

Our study preliminarily identified several factors associated with the development of MCI in hospitalized older adults with PD, including lower education levels, greater severity of motor symptom (as measured by MDS-UPDRS-III scores), anxiety, and reduced hemoglobin levels. Among these, lower educational attainment was linked to a higher risk of MCI, consistent with previous findings (Campos et al., 2024; Jia et al., 2020). Individuals with lower education may have fewer opportunities for cognitive stimulation and access to social support resources, both of which are known to increase vulnerability to cognitive decline (Lövdén et al., 2020; Wang Y. et al., 2023). Higher educational attainment is believed to enhance cognitive reserve, offering a protective effect against cognitive deterioration (Wang et al., 2025). In addition, those with more education are more likely to adopt healthier lifestyles, engage in meaningful social activities, and access healthcare services more effectively—factors that may collectively help reduce the risk of MCI (Bertola et al., 2021; Wang et al., 2021). These findings suggest that healthcare providers should pay special attention to hospitalized older adults with PD who have lower education levels, who represent a high-risk group. Early screening and timely cognitive interventions tailored to this population may help prevent the onset of MCI.

Our findings indicate a significant association between motor symptom severity, as measured by MDS-UPDRS-III scores, and the presence of MCI in hospitalized older adults with PD. This is consistent with prior studies reporting a close relationship between the severity of motor symptoms and cognitive decline in PD patients (Kwon et al., 2022; Monastero et al., 2018). Additionally, research has shown that compared to cognitively normal PD patients, those with PD-MCI experience significantly faster deterioration in all motor domains except for tremor (Qin et al., 2022). Severe motor symptoms in PD often reflect poorer cognitive performance and may result from shared pathophysiological mechanisms involving widespread neurotransmitter dysregulation (Aarsland et al., 2021).

Earlier studies also suggest a reciprocal relationship between motor and cognitive impairments, with baseline cognitive performance influencing motor prognosis and cognitive decline predicting faster motor symptom progression (Chung et al., 2021). Thus, motor symptom assessment plays a vital role in the early prediction and monitoring of cognitive impairment in PD. In clinical practice, hospitalized older adults with PD exhibiting rapid motor deterioration should be screened for cognitive impairment as early as possible to avoid delays in MCI identification and intervention. Moreover, for hospitalized older adults with PD diagnosed with MCI, nurses should collaborate with physicians to develop comprehensive rehabilitation plans that integrate cognitive training and motor interventions to optimize overall therapeutic outcomes.

Our study also found that anxiety is significantly associated with the occurrence of MCI in hospitalized older adults with PD. This finding aligns with previous research that has identified anxiety as a notable risk factor for MCI (Krell-Roesch et al., 2021; Toloraia et al., 2022). Meng et al. (2023) reported that PD patients with comorbid anxiety and depression demonstrated impairments in attention, memory, visuospatial abilities, and executive functions. A strong association exists between anxiety and PD-MCI, and the development of anxiety is closely linked to more extensive cognitive impairment (Wang H. et al., 2023). Although the underlying mechanisms between anxiety and cognitive dysfunction remain unclear, several studies have suggested connections between anxiety and biomarkers of cognitive impairment (Pink et al., 2022; Sun et al., 2023). From a neurochemical perspective, dysfunctions in the dopaminergic, noradrenergic, and serotonergic systems may contribute to both anxiety and cognitive deficits in PD patients (Joling et al., 2018). Additionally, the attentional control theory provides a psychological explanation: anxiety increases attentional bias toward perceived threats, thereby negatively impacting cognitive performance (Eysenck and Nazanin, 2011). In PD, frequent motor fluctuations (i.e., “on–off” phenomena) may be perceived as threat-related stimuli, exacerbating anxiety and leading to attention deficits and progressive cognitive decline (Forbes et al., 2021). Since cognitive impairment in PD is often difficult to reverse, whereas anxiety symptoms are potentially modifiable through appropriate treatment (Gonçalves and Byrne, 2012), timely identification and management of anxiety in hospitalized older adults with PD may reduce or delay the onset of cognitive impairment. Furthermore, identifying specific cognitive domains affected by anxiety may help guide targeted therapeutic strategies for PD patients with comorbid anxiety and cognitive dysfunction.

Our study found that lower hemoglobin levels were significantly associated with the presence of MCI in hospitalized older adults with PD. This finding is consistent with previous research (Qiang et al., 2023; Qin et al., 2019). Hemoglobin is the primary oxygen-carrying protein in red blood cells, and reduced levels can impair oxygen delivery to tissues—an effect particularly pronounced in the brain (Burtscher et al., 2021; Kung et al., 2021). Emerging evidence suggests that chronic hypoxia may alter the excitability and functional expression of iron channels, accelerating the formation of beta-amyloid plaques (Kung et al., 2021). Even mild reductions in hemoglobin concentration have been linked to declines in cognitive performance (Penninx et al., 2003). This may be due to limited tissue oxygenation over time, resulting in chronic cerebral hypoperfusion, oxidative stress, mitochondrial dysfunction, progression of white matter hyperintensities, abnormal protein aggregation, and neuronal damage—all of which contribute to cognitive impairment (Dong et al., 2025; Meng et al., 2020). In addition, studies have associated low hemoglobin levels with lobar microbleeds, cortical thinning in the occipital lobe and global cortex, and reduced nucleus accumbens volume. Low hemoglobin is also considered a marker of chronic inflammation, frailty, and poor overall health—conditions closely tied to cognitive decline (Tan et al., 2018). Therefore, routine monitoring of hemoglobin levels in hospitalized older adults with PD may help detect underlying anemia and serve as an early indicator of cognitive impairment. Nurses can play a critical role by assisting with nutritional assessments, implementing dietary interventions, and managing anemia to help maintain optimal hemoglobin levels and potentially slow cognitive deterioration.

Our study also found an association between MCI and prolonged hospitalization duration among hospitalized older adults with PD. However, due to the limitations of the cross-sectional design, a causal relationship cannot be established. However, previous studies have identified cognitive impairment and functional dependence as key risk factors for extended hospitalization (Bo et al., 2016). A prospective cohort study of hospitalized patients aged 65 and older found a strong correlation between the degree of cognitive impairment and hospitalization duration—the greater the cognitive impairment, the longer the hospitalization (Power et al., 2017). These patients often require longer recovery periods due to functional deficits, and factors such as unfamiliar hospital environments, secondary complications (e.g., infections, delirium, falls, and adverse drug events), and emotional distress can further prolong their hospitalization duration (Chinnappa-Quinn et al., 2020; Schattner, 2023). Cognitive deficits may also hinder a PD patient’s ability to manage their condition and maintain overall health, potentially exacerbating existing conditions or triggering new complications that extend hospitalization (Chandler et al., 2021). Compared to cognitively intact patients, those with PD-MCI typically have poorer functional status, higher levels of anxiety and depression, and reduced quality of life. They often struggle to understand medical situations and engage in care decisions, which may delay treatment progress (Moelter et al., 2016). In this context, systematic cognitive assessment and management during hospitalization — including early cognitive screening, targeted cognitive interventions, and enhanced health education — may help reduce hospitalization burden and improve overall patient outcomes.

## 5 Limitations

This study emphasizes the importance of routine cognitive screening in clinical practice. We observed a high prevalence of MCI among hospitalized older adults with PD in China, highlighting the need for early detection and timely intervention. Identification of high-risk patients—such as those with lower education levels, more severe motor symptoms, anxiety, or reduced hemoglobin levels—may facilitate tailored management, potentially shortening hospitalization duration and reducing healthcare burden.

Despite the valuable insights of this study, several limitations should be noted. First, MCI was diagnosed solely using the MMSE. Although education-adjusted cut-off points were applied to mitigate bias related to educational attainment, the limited sensitivity and specificity of the MMSE may have led to an underestimation of MCI prevalence. Moreover, while the MMSE and other neuropsychological assessment tools are widely used internationally, their validity may vary across different cultural and linguistic contexts. Future cross-cultural studies or clinical applications should consider appropriate adjustments and careful interpretation of these instruments to ensure accurate and reliable assessments. Second, this study was conducted at a single center using convenience sampling of hospitalized older adults with PD. This approach may limit the representativeness of the sample, and the findings should be generalized with caution. Future research involving larger, multicenter cohorts is warranted to validate and extend these results.

## Conclusion

In conclusion, this study identified a relatively high prevalence of MCI among hospitalized older adults with PD and several factors potentially associated with its occurrence, including lower educational level, greater severity of motor symptoms, anxiety, and reduced hemoglobin levels. PD-MCI was also found to be associated with longer hospitalization duration. These findings highlight the potential importance of early cognitive screening and timely management in clinical practice. Targeted assessment and intervention for high-risk patients may help improve patient outcomes and alleviate healthcare burden.

## Data Availability

The raw data supporting the conclusions of this article will be made available by the authors, without undue reservation.
